# Pontine hemorrhage as beginning of bipolar disorder or organic mania. A case report

**DOI:** 10.1192/j.eurpsy.2021.1643

**Published:** 2021-08-13

**Authors:** P. Coucheiro Limeres, A. Franco Soler, A. Cerame Del Campo, L. Amaya Lega

**Affiliations:** Psychiatry, Instituto Psiquiatrico José Germain, Leganés, Spain

**Keywords:** bipolar disorder, stroke, secondary mania

## Abstract

**Introduction:**

Published evidence describes the appearance of manic episodes in patients who suffer localized brain lesions with no prior psychiatric history.

**Objectives:**

A case report is presented alongside a review of the relevant literature regarding the relationship between Bipolar disorder and strokes.

**Methods:**

We present the case of a 54-year-old man who, after suffering a pontine hemorrhage, developed a depressive mood for which he was treated with Sertraline 50 mg. The following month the patient developed hypomanic mood, disinhibition, insomnia and megalomaniac ideation. He was treated with Risperidone 2 mg and the antidepressant was withdrawn. The symptomology disappeared shortly after but a few months later he developed a major depressive disorder (inhibition, ideas of ruin and guilt, low mood, decreased intake and daily activities…). He was treated again with antidepressants (Citalopram 30mg) and lithium was introduced in the absence of a total response.

**Results:**

Mania secondary to brain lesions has been observed in multiple studies, where an association is made mainly with lesions at the frontal, temporal, subcortical limbic brain areas and in lesions causing hypofunctionality on the right side. Most of the cases described occurred in male patients with no prior psychiatric record and with associated vascular risk.

**Conclusions:**

It is important to carry out an exhaustive medical history to be able to identify the cases of secondary mania so as not to ignore the underlying neurological condition in the approach.

**Disclosure:**

No significant relationships.

